# Applications of Induced Pluripotent Stem Cells in Studying the Neurodegenerative Diseases

**DOI:** 10.1155/2015/382530

**Published:** 2015-07-09

**Authors:** Wenbin Wan, Lan Cao, Bill Kalionis, Shijin Xia, Xiantao Tai

**Affiliations:** ^1^Department of Neurology, Zhongshan Hospital, Fudan University, Shanghai 200032, China; ^2^State Key Laboratory of Medical Neurobiology, Department of Neurobiology and Institutes of Brain Science, School of Basic Medical Science, Fudan University, Shanghai 200032, China; ^3^Department of Perinatal Medicine, Pregnancy Research Centre and University of Melbourne Department of Obstetrics and Gynaecology, Royal Women's Hospital, Parkville, VIC 3052, Australia; ^4^Shanghai Institute of Geriatrics, Huadong Hospital, Fudan University, Shanghai 200040, China; ^5^School of Acupuncture, Massage and Rehabilitation, Yunnan University of Traditional Chinese Medicine, Kunming 650500, China

## Abstract

Neurodegeneration is the umbrella term for the progressive loss of structure or function of neurons. Incurable neurodegenerative disorders such as Alzheimer's disease (AD) and Parkinson's disease (PD) show dramatic rising trends particularly in the advanced age groups. However, the underlying mechanisms are not yet fully elucidated, and to date there are no biomarkers for early detection or effective treatments for the underlying causes of these diseases. Furthermore, due to species variation and differences between animal models (e.g., mouse transgenic and knockout models) of neurodegenerative diseases, substantial debate focuses on whether animal and cell culture disease models can correctly model the condition in human patients. In 2006, Yamanaka of Kyoto University first demonstrated a novel approach for the preparation of induced pluripotent stem cells (iPSCs), which displayed similar pluripotency potential to embryonic stem cells (ESCs). Currently, iPSCs studies are permeating many sectors of disease research. Patient sample-derived iPSCs can be used to construct patient-specific disease models to elucidate the pathogenic mechanisms of disease development and to test new therapeutic strategies. Accordingly, the present review will focus on recent progress in iPSC research in the modeling of neurodegenerative disorders and in the development of novel therapeutic options.

## 1. Introduction

Human embryonic stem cells (ESCs) are undifferentiated cells derived from the inner cell mass of the blastocyst. ESCs are characterized by their ability to proliferate indefinitely without differentiating and by their capacity to differentiate into all embryo-derived cell lineages [1]. ESCs are widely used for many purposes such as gene targeting, cell therapy, tissue repair, and organ regeneration [2]. However, the use of ESCs is hampered by significant barriers including immune rejection, incorrect tissue regeneration, tumor formation, and ethical concerns relating to the destruction of human embryos [3, 4]. Recently, Takahashi and Yamanaka demonstrated a novel approach for the preparation of iPSCs, which have pluripotency potential very similar to that of ESCs, through reprogramming mouse fibroblasts back to an immature, pluripotent state by retroviral-mediated introduction and overexpression of the pluripotent transcription factors (TFs) [5]. Subsequently, Takahashi et al. independently reprogrammed human somatic cells into human iPSCs that were similar to human ESCs in morphology, proliferation, surface antigens, gene expression, epigenetic status of pluripotent cell-specific genes, and telomerase activity [6, 7].

The use of iPSCs is now permeating into many sectors of disease research. Patient sample-derived iPSCs can be used to construct patient-specific disease models to elucidate previously unknown pathogenic mechanisms of disease development and to test new therapeutic strategies [8–15].

Neurodegeneration is the umbrella term for the progressive loss of structure or function of neurons, including death of neurons. Incurable neurodegenerative disorders such as Alzheimer's disease (AD), frontotemporal dementia (FTD), amyotrophic lateral sclerosis (ALS), Parkinson's disease (PD), Huntington's disease (HD), and multiple sclerosis (MS) have reached a staggering prevalence [16]. Despite great progress in understanding the etiology of the disorders, the underlying mechanisms are still indistinct. Furthermore, no means of treating the underlying cause have been devised. Although transgenic and knockout models of neurodegenerative diseases are extensively employed and have yielded important insights into some molecular mechanisms of disease development, the models do not provide the opportunity to study mechanisms of neurodegeneration in human neurons at risk. Thus, it is often difficult or even impossible to replicate human pathogenesis with this approach [17]. The field is hindered by the paucity of human disease-specific models for the development of new drugs to control these diseases. For the past decade, researchers have been interested in stem cells and the prospect of using them for understanding the pathogenesis of disease and for facilitating the development of novel therapeutics [18–20]. Using disease-specific iPSCs derived from patients permits the preparation of neurons that contain the genetic information of the individual patients. Accordingly, the present review will focus on recent progress of iPSC research in the modeling of neurodegenerative disorders and in the development of novel therapeutic options.

## 2. Traditional Methods of Establishing Stem Cells

Stem cells are roughly categorized as ESCs, mesenchymal stem cells (MSCs), and iPSCs [21]. The reprogramming technology reverses differentiated somatic cells (e.g., skin fibroblasts and blood lymphocytes) into stem cells by epigenetic modification [5, 6, 22–25]. Until recently, resetting the epigenome of a somatic cell to a pluripotent state was achieved by somatic cell nuclear transfer (SCNT) [22], cell-cell fusion [23], treatment with extracts of pluripotent cells [24], and ectopic expression of defined factors [5, 6] (Table 1).

The first successful cloning experiments in mammals by introduction of nuclei of adult somatic cells into oocytes (SCNT technology) that gave rise to viable offspring clearly demonstrated that the cytoplasm of oocytes must contain sufficient genetic information to reprogram nuclei of at least some cell types [26]. However, the utilization of SCNT for research, or for potential stem cell-based therapies tailored to human patients, is restricted by low cloning efficiency and the observation developmental abnormalities in test animals at different stages of development [26, 27]. Moreover, ethical problems associated with deriving, and working on, large numbers of human oocytes have further restricted the application of SCNT in the clinic [26]. The creation of cell hybrids by fusion of somatic cells with pluripotent cells of various origins has successfully reprogramed somatic cells derived from mice or humans and generated pluripotent cells [28, 29]. However, the utility of this method is hindered because the resultant cell hybrids are tetraploid [23, 29], which may limit the use of this method for clinical applications. In addition, both SCNT and cell fusion entail complex mixtures of known and undefined factors from oocytes or pluripotent cells to trigger reprogramming, making mechanistic studies particularly challenging [26–28].

Incubation of reversibly permeabilized cells with the cell-free extracts of pluripotent cells, such as ESCs [24], embryonic germ cells [30], or* Xenopus* oocytes [31], also induces partial reprogramming of the nucleus. The reprogrammed cells reexpress pluripotency markers and redifferentiate into multiple lineages [24, 30, 31]. Compared with SCNT or cell fusion, this method does not introduce ESC chromosomes into somatic cells. However, the method is limited when applied to primary cells, since the reprogrammed cells regain only some pluripotent cell properties [26]. Furthermore, it has been suggested that in some cases the possibility cannot be excluded that the reported reexpression of pluripotency-associated genes is due to material from the pluripotent donor cells [26].

## 3. iPSC Technology

A new strategy was demonstrated by Takahashi et al. that avoided all the aforementioned issues, in which mammalian somatic cells were reprogrammed to iPSCs by ectopic expression of the pluripotent TFs* Oct4* (also known as* Pou5f1*),* Sox2*,* Klf4*, and* c-Myc* (known as* OSKM*) [5, 54]. In 2007, human iPSCs were first generated by the same group by transducing adult human dermal fibroblasts with viral vectors carrying the same TFs using a retroviral system [6]. The iPSCs generated in this manner share the main properties of preimplantation embryo-derived ESCs, which include unlimited self-renewal and the potential to differentiate into cells of the three germ layers [5, 6]. In the same year, Yu et al. also generated human iPSCs, in which the factors* Oct4*,* Sox2*,* Nanog*, and* LIN28* and a lentiviral system were used to reprogram human somatic cells to pluripotent stem cells that exhibit the essential characteristics of ECSs [7]. Takahashi and Yamanaka identified 24 genes as candidate factors to play pivotal roles in the maintenance of ESC identity, which were subsequently retrovirally transduced into Fbx15-receptor-fibroblasts, using an assay system where the induction of pluripotent state could be detected by resistance to G418 [5]. The 24 candidate factors were progressively reduced in number by stepwise exclusion of single factors until only four genes remained that were sufficient and necessary to induce iPSCs [5]. Despite the enormous potential for iPSCs in human disease research, there remain several limitations in applying iPSCs in the clinic. The slow reprogramming process and low reprogramming efficiency impede detailed mechanistic studies and potential applications of this technology. In the beginning, only about 0.1% to 1% of somatic cells experience changes at the transcription level and finally become pluripotent stem cells when methods are used that do not involve genome integration [37].

**Figure 1:**
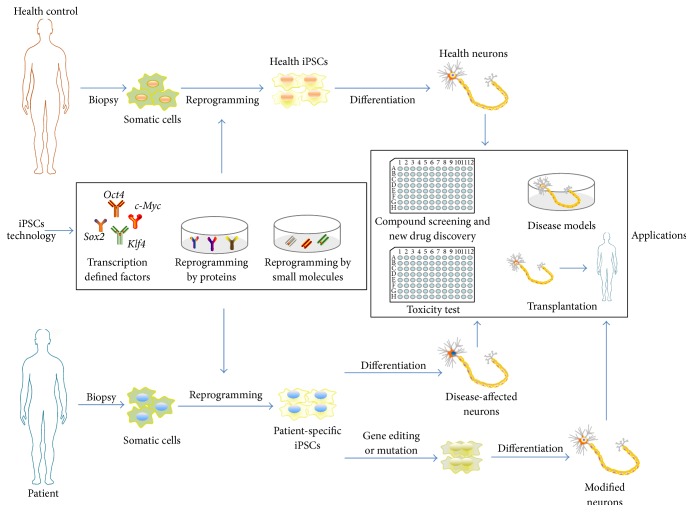
Basics of iPSC technology. Adult somatic cells can be reprogrammed into stem cell by the iPSC approach. iPSC-derived specific neurons can be used in drug screening and new drug discovery, toxicity tests, and models for studying the neurodegenerative diseases and for self-transplantation therapy.

In recent years, approaches have been continually refined and optimized to improve efficiency and increase the ratio of iPSC colonies to total colonies [55, 56]. However, the method employing the original 4 TFs [5, 6] remains the preferred option. Methods that have been explored to facilitate clinically relevant iPSC gene delivery include plasmids [57], piggyBac vectors [58], and minicircle vectors [59]. Although the iPSC reprogramming approach via viral vectors shows relatively higher reprogramming efficiency than nonviral delivery, the genome may be mutated by integrating other gene sequences, thus raising safety concerns [38]. Furthermore,* c-Myc *is an oncogene and this increases the risk of tumor formation [39]. Recent studies have successfully generated transgene-free and genetically flawless iPSCs using protein transduction [32, 33], nonintegrating viral vectors such as Sendai virus [60], episomal vectors [61], transfection of modified mRNA transcripts [62], and small molecules [34–36] (Figure 1). Adenovirus and Sendai virus transduction techniques avoid exogenous DNA integration into the host genome and are highly efficient but there is a tendency for cells to carry the virus genome. Thus, compared to the traditional viral methods, the nonviral approaches can be used to generate qualified iPSCs without the risk of insertional mutagenesis. However, outcomes from these methods have been mostly unsuccessful. Attempts at reprogramming with proteins have been successful but the efficiency of production is extremely low (~0.001%) [63].

An alternative to the generation of iPSCs is to generate the target cell of interest directly from differentiated cells. In an initial set of experiments conducted by Vierbuchen et al., fibroblasts were directly reprogrammed into induced neuronal cells (iNCs) by inducing the expression of neuronal lineage-specific TFs [64]. The combined expression of only three factors,* Ascl1*,* Brn2*, and* Myt1l,* was sufficient to rapidly and efficiently convert mouse embryonic and postnatal fibroblasts into functional neurons* in vitro *[64]. These iNCs express multiple neuron-specific proteins, generate action potentials, and form functional synapses [64]. Subsequent studies demonstrated that other neurogenic factors, namely,* Ngn2*,* Ascl1*, and* Dlx2,* can also reprogram early-postnatal astrocytes into neurons* in vitro*, including both GABAergic and glutamatergic fates [65–67]. Subsequently, diverse neurons such as the cholinergic [68] and the dopaminergic [69] neurons have been produced from many differentiated cell types including hepatocytes [70], pericytes [71], and adult astrocytes [72] but most often from fibroblasts [64]. These findings clearly show that the overexpression of a few “master” factors is sufficient to drive a relatively rapid, direct specific lineage change in cells derived from different embryonic layers. Generation of iNCs from nonneuron lineages could represent the starting point for using these cells in regenerative medicine and could also be useful for improving our knowledge about neural development and neurological disease pathogenesis [73].

## 4. Applications of iPSCs in Neurodegenerative Diseases

Through the establishment of iPSC technology, adult somatic cells can develop a pluripotent state [3–5, 74]. Indeed, human iPSCs have effectively bypassed the ethical constraints and this approach made it possible to produce iPSCs from biopsy samples of arbitrarily selected individuals and to subsequently maintain, expand, and stock these cells without concerns relating to race, genetic background, or state of health [8]. Furthermore, owing to their ESC-like pluripotency, iPSCs allow the production of a variety of derived cells such as neurons, not only for applications in regenerative medicine such as transplantation therapy but also for modeling human diseases and new drug development [8].

Neurodegenerative diseases occur when neurons begin to deteriorate. Changes in these cells cause them to function abnormally and eventually result in the demise of the cell. The incidence of these diseases is expected to rise dramatically as life expectancy increases, which represents a significant economic and social burden. Animal models (e.g., transgenic animals or knockout animals) of neurodegenerative diseases have been generated to understand disease mechanisms and to provide a platform for testing therapeutic strategies [8, 14, 17, 75, 76] but the construction of models that can accurately and thoroughly reproduce human pathology remains problematic. Furthermore, due to species variation and differences in cell line specificity, there is substantial debate as to whether animal and cell line disease models accurately reflect the natural phenomena that occur in human patients [8]. Excitingly, disease-specific iPSCs have been established from patients with neurodegenerative diseases, thus opening research avenues for studying underlying mechanisms and exploring therapies. Recently, human iPSCs derived from patients with the neurodegenerative diseases including ALS, PD, AD, and SMA have been successfully differentiated* in vitro* into disease-relevant cell types, including motor neurons (MNs), dopaminergic neurons, and oligodendrocytes [77] (Table 2).

### 4.1. Alzheimer's Disease

AD is the most common form of senile dementia and is characterized by senile plaque (SP) formation, which is composed of extracellularly deposited *β*-amyloid (A*β*) and also neurofibrillary tangles (NFT) that contain tau, which is an intracellular aggregated, hyperphosphorylated protein that belongs to the microtubule-associated protein (MAP) group [78]. The pathogenesis of AD has been intensively studied in the last decade [78, 79]. However, the mechanisms underlying the neuron defects and synapse damage in AD are still unclear and currently there are no effective therapies for AD. Fortunately, the recent development of an AD disease model using iPSCs provides access to cell types that were previously unobtainable in sufficient quantity or quality and this presents exciting prospects for elucidating the etiology of AD and for the development of potential therapeutics [20]. Several groups have since generated neurons from human iPSCs carrying mutations in genes encoding amyloid precursor protein (APP) and presenilin (PS), demonstrating that they recapitulate the APP processing pathway and provide an innovative approach to studying the pathogenesis of AD [11, 80, 81].

Kondo et al. derived human iPSCs from atypical early-onset familial AD (fAD) patients with APP-E693Δ mutation showing overt early-onset symptoms of AD but lacking A*β* deposition [82], as well as from sporadic AD (sAD), and differentiated them into neural cells [11]. They found that A*β* oligomer accumulation led to endoplasmic reticulum (ER) and oxidative stress [11]. Furthermore, the accumulated A*β* oligomers were not proteolytically resistant and subsequent treatment with docosahexaenoic acid (DHA) alleviated the stress responses in the AD neural cells [11]. These results may explain the variable clinical results obtained with the use of DHA treatment and suggest that DHA may actually be effective for a specific subset of patients [11]. Indeed, human iPSCs-derived neurons displayed elevated levels of A*β*_1–42_ that responded to treatment with *γ*-secretase inhibitors [81] and these results supported previous observations regarding the effect of PS mutations [83].

In another study, *β*-secretase inhibitors were investigated in iPSCs-derived, purified neurons from patients with fAD caused by a duplication of the APP gene (*APP*; termed APP^Dp^) and sAD [80]. Compared to nondemented controls, the neurons from the APP^Dp^ patients exhibited significantly higher levels of the pathological markers A*β*_1–40_, phospho-tau (Thr 231), and active glycogen synthase kinase-3*β* (aGSK-3*β*) [80]. Treatment of the purified neurons with *β*-secretase inhibitors caused a significant reduction in phospho-tau (Thr 231) and aGSK-3*β* levels [80]. These results suggest a direct relationship between APP proteolytic processing, but not A*β*, in GSK-3*β* activation and tau phosphorylation in human neurons [80].

### 4.2. Parkinson's Disease

PD is the second most common chronic progressive neurodegenerative disorder. The clinical features of PD, which are characterized by combinations of motor problems, namely, bradykinesia, resting tremor, rigidity, flexed posture, “freezing,” and lose of postural reflexes, most likely result from the loss of dopaminergic (DA) neurons in the* substantia nigra pars compacta* [84, 85]. Our understanding of the underlying molecular mechanism of PD is hampered by restricted access to affected human DA neurons on which to base experimental research. The majority of PD cases are sporadic, with up to 20% of patients presenting with familial monogenic forms of the disease [43]. Patient-specific iPSCs from idiopathic PD cases allow the generation of midbrain DA neurons that have the same genetic composition as the patients and they share many important properties with the nigral DA neurons in the PD patients [44]. Furthermore, the iPSC-derived midbrain DA neurons can be transplanted into the adult rodent stratum, where they show arborization and mediate functional effects as determined by the reduction of amphetamine- and apomorphine-induced rotational asymmetry [86]. However, only a few DA neurons projected into the host striatum at 16 weeks after transplantation [86].

Although monogenic forms of PD only account for a small percentage of PD cases [87], understanding how mutations of these genes cause the degeneration of DA neurons is critically important for the study of the disease mechanism and for the identification of disease-modifying drugs. Mutations in* GBA*,* LRRK2*,* PARK2*,* PARK7 *(*DJ-1*),* PINK1*,*α-Synuclein* (*SNCA*), or* UCHL1* can lead to monogenic forms of PD or increased PD susceptibility, suggesting important roles for these proteins in the pathogenesis of the disease [43].* SNCA *is the first gene linked to autosomal-dominant familial PD, which encodes a synaptic vesicle-associated protein that appears in high abundance in Lewy bodies [44]. In a recent study, iPSCs were derived from a PD patient with triplication of the* SNCA *gene [88]. Compared with the normal controls derived from an unaffected first-degree relative, DA neurons derived from this patient produced double the amount of *α*-Synuclein protein [88]. Mutation of the* LRRK2 *gene is the most common PD-related mutation [44, 89]. iPSCs carrying the* p.G2019S *mutation (G2019S-iPSCs) in the* LRRK2 *gene were able to differentiate into DA neurons and showed increased expression of key oxidative stress response genes, as well as upregulation of *α*-Synuclein protein [89].

*PINK1 *is a gene that encodes a mitochondrial kinase, which protects cells against mitochondrial stress and regulates mitochondrial degradation [90]. Loss of* PINK1 *has been linked to increased levels of oxidative stress [91]. Seibler et al. reported that fibroblasts from genetic PD with* PINK1 *mutations can be reprogrammed and differentiated into dopaminergic (DA) neurons [85]. The neurons, upon mitochondrial depolarization, showed impaired recruitment of lentivirally expressed* Parkin* to mitochondria, increased mitochondrial copy number, and upregulation of the important regulator of mitochondrial biogenesis, PGC-1*α* [85]. Importantly, these alterations were corrected by lentiviral expression of wild-type* PINK1* in mutant iPSC-derived* PINK1 *neurons [85].

### 4.3. Amyotrophic Lateral Sclerosis

ALS is an adult-onset neurodegenerative disease that involves the upper and lower MNs from the motor cortex, brain stem, and spinal cord and leads to muscle denervation, wasting, and death [92]. There are no effective cures for ALS, though the benzothiazole riluzole slows the rate of progression and prolongs survival by three months [93]. About 10% of ALS cases are familial (fALS) and are caused by mutations in one of at least 32 known genetic loci, which include superoxide dismutase 1 (*SOD1*) [94], Tar DNA binding protein-43 (*TDP-43*) [95], fused in sarcoma (*FUS*) [47], and* C9ORF72* [48]. Sporadic ALS (sALS) accounts for 90% of cases, and in these cases the genetic etiology is largely unknown [96]. Recently, ALS-disease models were developed that employ patient-derived iPSCs to explore the pathogenesis of ALS and to test drug strategies [97–99].

Mutations in* SOD1* are the most studied mutations related to ALS [94]. Researchers created iPSC-derived MNs from patients with the most common North American ALS genotype, A4V* SOD1*, as well as D90A* SOD1* [100]. Neurofilament (NF) aggregation followed by neurite swelling appeared early in spinal MNs but rarely in non-MNs [100]. These changes were associated with binding of mutant* SOD1* in the 3′ UTR of* NF-L* mRNA, decreased stability of* NF-L* mRNA, and thus altered proportion of NF subunits [100]. Moreover, such MN-selective changes were mimicked by the expression of a single copy of the mutant* SOD1* in human ECs and were prevented by genetic correction of the* SOD1* mutation in patient's iPSCs [100]. Importantly, conditional expression of NF-L in the* SOD1* iPSC-derived MNs corrected the NF subunit proportion, mitigating NF aggregation and neurite degeneration [100]. Taken together, these data suggest that NF misregulation underlies mutant* SOD1*-mediated NF aggregation and axonal degeneration in ALS MNs.

TDR43 is found in cytoplasmic inclusions in 95% of ALS, and about 4% of familial ALS is caused by mutations in TDP-43 [96]. Genetic analyses identified more than 30 mutations in the TDP-43 gene in both familial and sporadic ALS cases [97]. Egawa et al. found that iPSCs-derived MNs from ALS patient carrying mutations in TDP-43 formed cytosolic aggregates similar to those seen in postmortem tissue from ALS patients and exhibited shortened neurites, as observed in a zebrafish model of ALS [99]. Currently, more than 30 different mutations, including the M337V mutation, have been described in ALS patients [96]. MNs derived from M337V-TDP-43-iPSCs were more susceptible to cellular stress and showed increased vulnerability in a variety of* in vitro* culture assays [98, 99].

### 4.4. Spinal Muscular Atrophy

SMA is among the most common genetic neurological diseases that cause infant mortality [101] and is characterized by the degeneration of spinal MNs and muscle atrophy [102]. Clinically, patients with SMA typically show symptoms at 6 months of age and die by age 2 [103]. Although the genetic cause of SMA has been mapped to the survival motor neuron 1 (*SMN1*) gene, which results in selective degradation of MNs, the mechanisms underlying selective MN degeneration in SMA remain largely unknown [102].

Ebert et al. reported the generation of iPSCs from skin fibroblast samples taken from a child with SMA and subsequently the iPSCs were differentiated into MNs, which expressed particular defects when compared to MNs from the child's unaffected mother [103]. SMA-derived iPSCs treated with valproate or tobramycin showed a significant increase in SMN levels compared with untreated iPSCs [103]. Furthermore, Chang et al. derived iPSCs from the fibroblasts of SMA patients and noted that SMA-iPSCs exhibited a reduced capacity to form MNs and aberrant neurite outgrowth [104]. Ectopic SMN expression in these iPSC lines restored normal motoneuron differentiation and rescued the phenotype of delayed neurite outgrowth [104]. These comprehensive data suggest that the observed abnormalities are indeed caused by SMN deficiency and not by iPSC clonal variability.

Corti et al. generated iPSCs from skin fibroblasts derived from SMA patients using nonviral, nonintegrating episomal vectors and used a target gene correction approach based on single-stranded oligonucleotides to convert the* SMN2* gene into an* SMN1*-like gene [101]. MNs formed by differentiation of SMA-iPSCs reproduced disease-specific features that were ameliorated in MNs derived from genetically corrected SMA-iPSCs [101]. Furthermore, the transplantation of corrected MNs derived from SMA-iPSCs into an SMA mouse model extended the life span of the animals and improved the disease phenotype [101].

### 4.5. Down Syndrome

DS, or trisomy 21 (T21) syndrome, is one of the most common chromosomal abnormalities and is characterised by mental retardation, cognitive impairment, and deficits in learning and memory. The syndrome is caused by an extra duplication of chromosome 21, which harbors miRNAs including miR-99a, miR-155, and miR-802 [51]. The brains with DS also display many neuropathological features including alterations in neurogenesis and synaptogenesis and early onset of AD-like symptoms [51]. Adults with Down syndrome develop early-onset AD, most likely due to increased expression of a gene on chromosome 21 that encodes the APP [105]. Shi et al. found that cortical neurons generated from DS patient-derived iPSCs produced the AD pathogenic peptide fragment A*β*_42_, which formed insoluble intracellular and extracellular amyloid aggregates and hyperphosphorylated tau protein in cell bodies and dendrites [105].

Other researchers were able to model neurogenesis impairment in DS with iPSCs derived from T21 amniotic fluid cells (AF) through lentiviral delivery and their subsequent differentiation into neuronal progenitor cells (NPCs) [106]. T21 AF-iPSCs were characterized by the expression of pluripotent markers and for their ability to differentiate into all three germ layers by forming embryoid bodies* in vitro*, and teratomas* in vivo*, as well as by their unique chromosomal karyotypes: three pairs of chromosome 21 [106]. However, the T21 AF-iPSC-NPCs generated fewer neurons compared with controls and exhibited developmental defects during neurogenesis [106]. In DS neurons, overexpression of miR-155 and miR-802 inhibited the expression of the target, methyl-CpG-binding protein 2 (MeCP2) [107]. In T21 AF-iPSC-NPCs, the investigators found that the expression levels of miR-155 and miR-802 were highly elevated and there was low expression of methyl-CpG-binding protein 2 (MeCP2) and thus this reflected the observations in DS neurons [106].

### 4.6. Polyglutamine Disease

Polyglutamine diseases comprise a family of neurodegenerative conditions that arise from a CAG triplet repeat expansion in a specific gene [108]. This expansion culminates in the expansion of a pathogenic protein containing a critically expanded tract of glutamines, which alters protein folding [108]. Protein misfolding disorders include HD, spinobulbar muscular atrophy, dentatorubral pallidoluysian atrophy, and several spin cerebellar ataxias [108]. iPSC studies have thus far been primarily focused on HD [109–111]. HD is a genetically dominant neurodegenerative condition characterized by progressive loss of motor and cognitive function that is caused by degeneration of selected neuronal populations within the basal ganglia and the cerebral cortex [112]. The pathology of HD is mainly driven by trinucleotide repeat expansion (CAG) (>39 CAG repeats manifest in disease) on chromosome 4 that results in an expanded polyglutamine tract at the encoding site of huntingtin protein (HTT) [53, 113]. iPSCs-based models from HD patients carrying a CAG repeat expansion were originally developed by Zhang et al. [109]. They found that the iPSCs-derived neurons showed enhanced caspase activity upon growth factor deprivation compared with normal cells [109]. Expansion of the polyglutamine tract in the huntingtin protein results in massive cell death in the striatum of HD patients. Using human iPSCs derived from HD patient fibroblasts, An et al. detected the expanded polyglutamine by the using of disease-specific antibody 1C2 that recognizes the expanded polyglutamine stretch [110]. Another study showed that transplantation of neural precursors derived from HD patient-specific iPSCs carrying 72 CAG repeats (HD72-iPSCs) into YAC128 mice significantly improved the behavior of the grafted mice [111]. Interestingly, the transplanted HD72-iPSC-derived neural precursors formed GABAeric neurons efficiently, but no EM48-positive protein aggregates were detected after transplantation [111].

## 5. Application in Studying Therapeutics

Owing to the inherent disconnect between drug pharmacology in heterologous* in vitro* cellular models and drug efficacy* in vivo*, the quest for more predictive* in vitro* systems is one of the most urgent challenges of modern drug discovery [76]. One reason for the failure of many drug candidates in humans is the poor predictivity of preclinical biological models [76]. Improved pharmacological* in vitro* profiling would require primary samples of the proper drug-targeted human tissue or* bona fide* human disease-relevant cells [76]. With the advent of iPSC technology, ready access to a variety of disease-relevant target cells is now within reach. The technology allows cells obtained directly from patients with neurodegenerative diseases to be propagated indefinitely and differentiated into the susceptible neuronal subtypes, offering the promise of discovering unique, human-specific disease models [3, 73, 77].

Genetic susceptibility to disease is a feature of most neurodegenerative disorders, and since patient-derived iPSCs carry the genetic background of the donor, this enables accurate modeling of genetic diseases* in vitro* [44]. Thus, personalized medicine such as specific drug treatment and cell-based transplant with required tissues can be conducted using patient-derived disease-specific iPSCs. To examine* in vivo *function of protein-based human iPSC-derived NPCs and/or DA neurons, transplantation studies were carried out in a well-established rodent model of PD with striatal lesions [114]. As determined by amphetamine-induced rotation scores, high concentration cell solution of Pro-NPCs but not the cells of terminal differentiation resulted in a dramatic functional recovery in parkinsonian rats [114], supporting the clinical potential of iPSCs for personalized cell therapy application. Furthermore, iPSCs can also be applied to chemical library screening for drug discovery [99, 115], as well as to subsequent testing for drug toxicity and efficacy [116]. For example, neurons derived from human iPSCs were firstly determined to be vulnerable to A*β*_1–42_ aggregates [116]. Then, by using the iPSCs, the investigators screened a chemical library containing several hundred compounds and discovered several small molecules, such as GW8510, as effective blockers against A*β*_1–42_ toxicity [116]. These pave the way for streamlined, economic, and efficient drug discovery research and improved drug discovery.

The long-term goal of iPSC-based strategies is to create patient-specific donor cells for transplantation therapy, thereby overcoming the ethical issues and the lack of tissue and cell availability, whilst avoiding immunorejection, which is a major complication in current transplantation medicine [4]. Several studies report transplantation of iPSCs into neurodegenerative disease models, particularly rodent models [86, 114, 117, 118]. An early study showed that neuronal cells derived from human iPSCs could be transplanted into the fetal mouse brain [117]. Another study showed that PD-derived iPSCs, which were differentiated into dopamine neurons, could be transplanted into the adult rodent striatum where some cells developed axons projecting into the striatum [86]. 6-Hydroxydopamine- (6-OHDA-) lesioned rats transplanted with the iPSC-generated neurons showed reduced amphetamine- and apomorphine-induced rotational asymmetry [86]. Nizzardo et al. found ALS mice treated by systemic administration of neural stem cells (NSCs) derived from ALS patient iPSCs exhibited improved neuromuscular function, motor unit pathology, and significantly increased life span when compared with control ALS mice [118]. Another study also showed that transplantation of human protein-based iPSCs (i.e., derived without any viral or other DNA-based vectors) into rats with striatal lesions could rescue motor deficits [114].

## 6. Conclusions and Future Directions

In this review, we focused our attention on neurodegenerative disease-specific iPSCs and described the current status of research in the field. As discussed above, iPSC researchers have developed new strategies to study the pathophysiology of human diseases and have provided assay systems for drug screening, as well as for regenerative medicine. The modern technique to generate personalized iPSCs changes our way of thinking regarding the exploration of disease pathogenesis and therapy development. Our understanding of the pathogenesis of neurodegenerative diseases is currently limited by difficulties in obtaining live neurons from patients and the inability to model the sporadic forms of the disease. Reprogramming adult somatic cells from patients into iPSCs and neurons may overcome these difficulties.

Current research indicates that neurodegenerative disorder-specific iPSC technology will accelerate the testing of small molecule therapeutics and cell transplantation therapy before the onset of clinical symptoms, or during the initial stages of the disease, so that therapeutics can reach human patients more quickly. However, there continue to be some key issues that must be addressed in the near future before the clinical application of iPSCs. These issues include generating homogenous populations of iPSCs and developing efficient methods of inducing and authenticating target cell types. Despite great promise and intense research into iPSC technology, iPSCs have not yet saved patient lives. Continuous interaction between researchers in the fields of basic stem cell biology, clinical investigation of diseases, translational research, pharmaceutical science, regulatory science, and system biology will be essential to ensure the potential of iPSCs contributing significantly to human health comes to fruition. iPSCs are in an early, but exciting and promising, stage of development but there remain many issues that need to be addressed before utilization in the clinic.

## Figures and Tables

**Table 1:** Approaches for establishing stem cells.

Strategy	Principle	Limitations
Somatic cell nuclear transfer	The earliest example of experimentally induced reprogramming that involves the transfer of a somatic nucleus into an enucleated oocyte to produce cloned animals [22, 25].	Low cloning efficiency, ethical problems, and observed abnormalities at different stages of development in test animals [26, 27].

Cell-cell fusion	Hybrid cells created by fusion of either mouse or human pluripotent cells with somatic cells, resulted in the reprogramming of the somatic genome to an embryonic state. The hybrid cells retained the characteristics of pluripotent cells [28, 29].	Hindered by the resultant cell hybrids that are tetraploid [23, 29].

Exposed to extracts of pluripotent cells	Incubation of reversibly permeabilized cells with the cell-free extracts of pluripotent cells such as ESCs [24], embryonic germ cells [30], or *Xenopus* oocytes [31].	The reprogrammed cells regain only some properties of pluripotent cells. We cannot properly exclude the possibility that the reexpression of pluripotency properties is due to material from the pluripotent cells in some cases [26].

Induced pluripotent stem cells	Reprogramming somatic cells back to an immature, pluripotent state by introduction of the pluripotent transcription factors [5, 6], by using protein transduction [32, 33], or by incubation with small molecules [34–36].	Slow reprogramming process and low reprogramming efficiency [37]. The viral vectors may integrate into cell genome and in particular the oncogene *c-Myc* increases the risk of tumor formation [38, 39].

**Table 2:** Associated genes in patients with neurodegenerative diseases.

Disease types	Genes	References
Alzheimer's disease	*APP*,* PS1*, *PS2*, *APOE *	[40–42]

Parkinson's disease	*GBA, LRRK2, PARK2, PARK7, PINK1, SNCA*,* UCHL1*, *MAPT*	[43–45]

Amyotrophic lateral sclerosis	*SOD1*, *FUS*, *TDP-43*, *C9ORF72 *	[46–48]

Spinal muscular atrophy	*SMN1*, *SMN2 *	[49, 50]

Down syndrome	*Trisomy 21 *	[50, 51]

Huntington disease	*HTT *(CAG-type repetitive DNA sequences)	[52, 53]
